# Study on the linkage between macro policy and market effectiveness in China’s stock market: Based on run test of China’s stock market index

**DOI:** 10.1371/journal.pone.0281670

**Published:** 2023-02-27

**Authors:** Manqing Liu, Shiting Ding, Qintian Pan, Yanming Zhang, Jingru Zhang, Qiong Yang, Tongtong Fang

**Affiliations:** 1 Nanjing Audit University Jinshen College, Nanjing, Jiangsu, P. R. China; 2 School of Economics and Management, Anhui Agricultural University, Hefei, Anhui, P. R. China; Universiti Malaysia Sabah, MALAYSIA

## Abstract

The macro policy of the stock market is an important market information. The implementation goal of the macro policy of the stock market is mainly to improve the effectiveness of the stock market. However, whether this effectiveness has achieved the goal is worth verifying through empirical data. The exertion of this information utility is closely related to the effectiveness of the stock market. Use the run test method in statistics to collect and sort out the daily data of stock price index in recent 30 years, the linkage between 75 macro policy events and 35 trading days of market efficiencies before and after the macro event are tested since 1992 to 2022. The results show that 50.66% of the macro policies are positively linked to the effectiveness of the stock market, while 49.34% of the macro policies have reduced the effectiveness of the market operation. This shows that the effectiveness of China’s stock market is not high, and the nonlinear characteristics are obvious, so the policy formulation of the stock market needs further improvement.

## Introduction

China’s stock market has undergone more than 30 years of development and become the second largest stock market in the world, basically matching the size of the Chinese economy. Although most of the early listed companies in China’s stock market were state-owned enterprises formed during the planned economy, the Chinese government has been trying to promote the marketization of its stock market [1]. In order to promote the market-oriented level of stock market, the Chinese government has been committed to continuously adjusting and influencing China’s stock market through macro policies in terms of mechanism design, market regulation and investor protection [2]. Since 1992, the Chinese government has introduced a large number of important macro policies affecting the development of the stock market, and such policy actions have made the Chinese stock market known as the "policy market". The question then arises whether these stock market macro policies introduced by the Chinese government have improved the marketability of the stock market or not? This is both a practical question to be assessed and a topic worth exploring at the academic level.

Macro policies play an important role in the operation of China’s stock market and are an important trigger for bull and the bear market transitions in China’s stock market [3]. To the best of our knowledge, academic research on the impact of macro policies on the Chinese stock market mainly includes the following aspects:

Firstly, the effects studies of macro policies based on changes in stock market performance. A study found that certain policy implementation in the Chinese stock market in the summer of 2015 did not have the desired effect by analyzing the effect of the policy, which illustrates the difference between the original intent and the outcome of stock policies [4]. It is also found that uncertainty policy instruments do not have a long-term impact on the Chinese stock market through the GARCH-MIDAS regression method [5], meanwhile there is no more profound impact on the Chinese stock market than the monetary policy of the People’s Bank of China [6]. However there is also evidence of the impact of the Chinese government’s uncertain macroeconomic policies on the Chinese stock market. This shows that studies on the performance of macro policies on stock market performance still do not reach consistent conclusions [7].

Secondly, the studies of the correlation between specific macro policies and stock market movements is also a major topic of academic research. From a macro perspective, market correlation increases when the market index falls down [8], and this correlation is not only seen in the Chinese stock market, the US stock market also shows a linkage between stock market indices and economic policies [9]. The correlation between macro policies and stock market indices in the Chinese stock market is not only in terms of the impact of policies on the stock market, but in essence, the extent of this correlation should actually be the stability of the stock market [10]. Further research shows that the correlation and two-way influence between Chinese stock market operations and relevant Chinese macro policies exhibit a long-term relationship [11]. By combing through these findings, it can be found that the linkage between macro policy and stock market in China is relatively significant and real.

Thirdly, relevant studies have analyzed the quantitative relationship between China’s stock market and its macro policies from the perspective of mathematics, and found a linear or nonlinear relationship between them. For example, there are research findings show that there are dynamic asymmetric spillovers and volatility correlations in China’s stock market [12], as for this volatility correlation, it has also been confirmed by relevant studies using the Granger Causality Test [13]. Global Economic Policy Uncertainty (GEPU) also has been found its effects on the volatility of the Chinese stock market [14].

The above researches show that China’s stock market macro policies may significantly affect the stock market trend, and the significant correlation between these macro policies and stock market changes has been confirmed by mathematical methods [15, 16]. The existing studies either interpret the relationship between the macro policies of China’s stock market and the operation of the stock market from an empirical perspective or from a phenomenological perspective [17]. However, as far as we know, too much research focuses on the objective and realistic relationship between the macro policies of China’s stock market and the operation of the stock market. The research on whether the macro policies of China’s stock market have changed the operation performance of the stock market is still insufficient. Since the starting point of macro policy making in China’s stock market is to improve the operating performance of the stock market, has the origin of such policy making been reflected in the operation of the stock market? It is worthwhile to analyze the actual implementation effects of these macro policies, especially from a long-term perspective, to determine whether these macro policies have achieved the purpose of improving stock market performance.

The Efficient Market Hypothesis (EMH), believes that if a stock market has sound laws, good functions, high transparency and sufficient competition, unless there is market manipulation, investors cannot obtain excess profits higher than the market average by analyzing past prices [18]. After the efficient market hypothesis was put forward, some classical studies found that the market efficiency of important stock markets represented by Asia-Pacific Markets stock market mostly hovered in the stage of weak-form efficient market [19]. Macro policy information in the stock market, as a kind of public information, plays its role on the premise that the stock market has a certain degree of effectiveness. However, the effectiveness of China’s stock market has been in a low and unstable state for a long time [20, 21], the linkage between stock market efficiency and market information presents a chaotic state [22].

If we review the evolution of China’s stock market, this so-called "chaotic state" stems from the "trial and error" of China’s macro policies. Similar to the reform process in other areas of China, the macro policies of the stock market are the most important means of the reform of the stock market, but also a gradual exploration of "crossing the river by feeling the stones" [23]. Whether the "trial and error" of the macro policy of the stock market will affect the positive effect of the macro policy information needs to be analyzed and studied through scientific means, and the efficiency of the stock market (Base on EMH) is an important indicator to measure the degree of marketization of the stock market [24].

Although there are many studies on the effectiveness of the stock market, few scholars use the effectiveness of the stock market to quantitatively analyze the implementation effect of the macro policy represented by the macro policy information. Especially in the process of the gradual evolution of the effectiveness of China’s stock market, whether the China’s stock market macro policies improved the effectiveness of the market? In other words, whether the macro policy information is effectively linked with the effectiveness of the stock market? In order to find more powerful evidence for these problems, this paper analyzes the linkage process between the effectiveness of China’s stock market and macro policy information, and provides data support and decision-making reference for the future macro reform of the stock market from the perspective of historical evolution.

## Theoretical analysis and research hypothesis

The essence of macro policy is a kind of market information, that is, exogenous market information, which is an important means to test the effectiveness of the stock market. To study investors’ reaction to macro policies, we can regard macro policies as an information flow from the perspective of investors, starting with the rational expectation hypothesis and efficient market theory of traditional finance. Rational expectation, also known as reasonable expectation, was originally proposed by J.F. Moose, an American economist. It can be understood that it is reasonable for rational investors to make predictions by using known information as much as possible. As rational people, they can make judgments, analyses, decisions and actions by using known information as much as possible. Therefore, in general, expectations made by people should be accurate. The concept of efficient market was put forward at the beginning of the 20th century. On this basis, American financial scientist Fama (1970) deepened and put forward the "efficient market hypothesis" [25]. For this hypothesis, it can be generally understood that when the information obtained by investors can fully reflect in the price, such a market is an efficient market. We can see from its assumptions that the conditions are contrary to reality in most cases, for example, people are not always risk averse.

**Fig 1:**
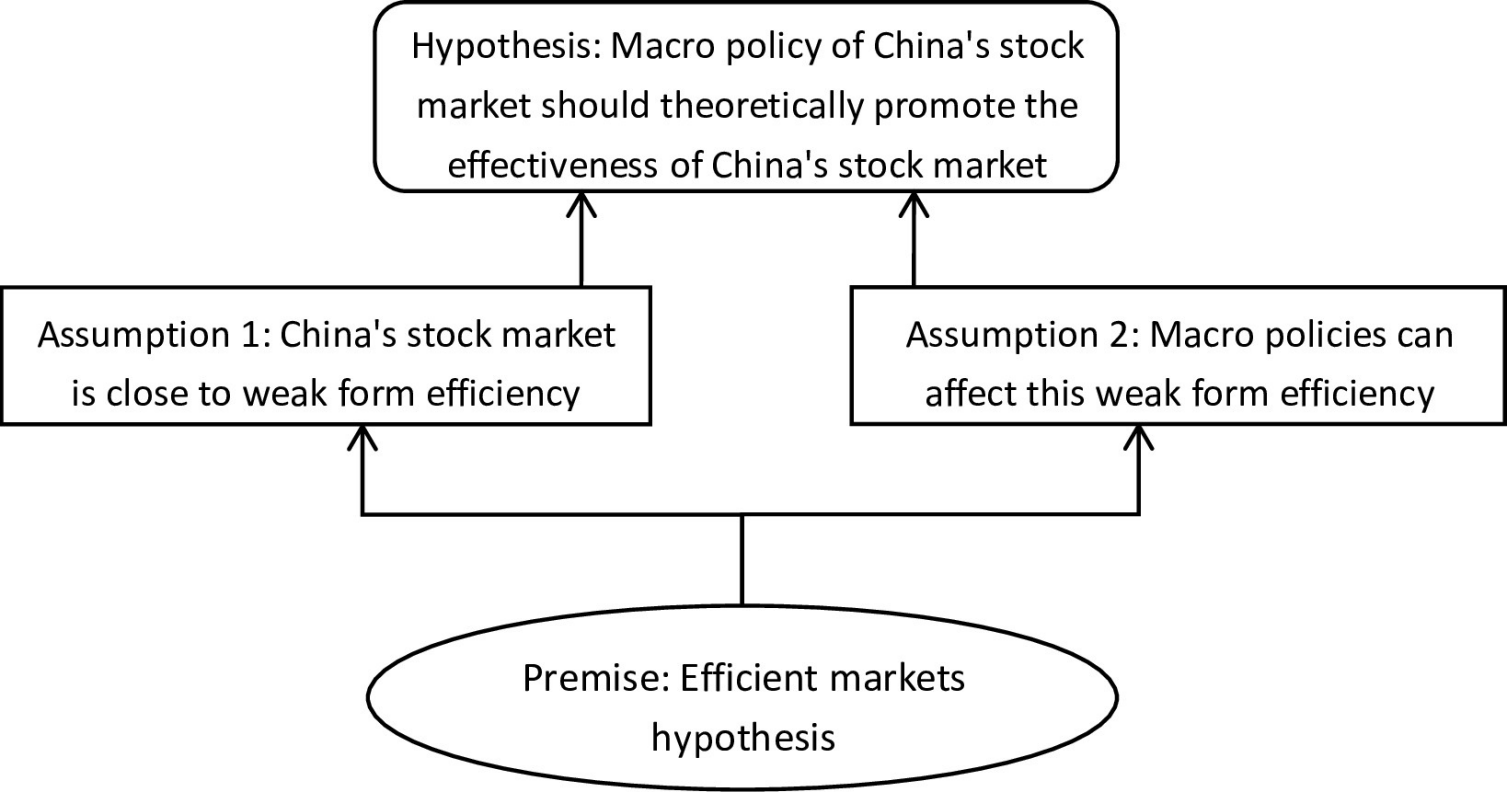
Concept and thought map.

First of all, this paper takes the efficient market hypothesis as the premise, and believes that the effectiveness of the market can reflect the soundness of the stock market to a certain extent [26]; secondly, based on this premise, this paper puts forward relevant assumptions, and believes that China’s stock market is close to weak form efficiency market, and macro policies can affect this weak form efficiency to a certain extent [27]; finally, it is assumed that the macro policy of China’s stock market should theoretically promote the effectiveness of China’s stock market, and this issue is also the core of the empirical test in this paper. The research idea is shown in the Fig 1.

Premise: Efficient markets hypothesis (EMH). If the price in a stock market fully reflects all available information, then such a market is called an efficient market. Under the weak form of efficiency, the market price has fully reflected all historical securities price information, including the transaction price and volume of stocks, short selling amount, financing amount, etc [28].

Assumption 1: The state of China’s stock market is between non weak efficient state and weak efficient state. Through the analysis of the historical data of China’s stock market, relevant research shows that part of China’s stock market has reached the weak efficient state, but many periods have not reached the weak efficient state [29, 30].

Assumption 2: The macro policy of the stock market is equivalent to an important kind of information. The impact of this information on the effectiveness of the stock market can last for a period of time [31]. During this period, it can be considered that the effectiveness of the market is affected by this information. Relevant researches shows that the average duration of the impact of important macro policy information on the entire stock market is about 30 to 40 trading days [32, 33]. This paper takes the median value for analysis, so 35 trading days are taken below.

Hypothesis: The macro policy of the stock market should promote the development of the effectiveness of China’s stock market towards enhancement, but it needs quantitative verification through technical analysis. The fundamental purpose of the macro policy is to improve and cultivate the stock market, especially the degree of marketization, so the introduction of macro policy should have a positive impact on the future market effectiveness [34]. However, due to the "chaotic state" of China’s stock market effectiveness, the linkage between the macro policy information of the stock market and the market effectiveness needs to be analyzed and tested.

## Materials and methods

### Selection and sorting of macro policy events

In order to study the impact of macro policies in the stock market on the effectiveness of China’s stock market, we first need to reasonably select the macro policies to be studied. According to the Delphi method [35], based on the principle of whether it belongs to macro policies and whether it belongs to major events, we sort out the policies that have been recognized as having significant impact on the stock market since its establishment 31 years ago as the research object. The results are listed in Table 1.

**Table 1:** Macro-policy information in China’s stock market (1990–2022).

No.	Date	Macro policy events
1	1992-02-19	China’s first economic regulations on joint-stock ownership of enterprises promulgated
2	1992-05-15	NDRC Document [1992] No.23
3	1992-05-21	Shanghai Stock Exchange fully opens the stock price
4	1992-10-26	The Securities Commission of the State Council were established
5	1992-12-17	NDRC Document [1992] No.68
6	1993-04-22	Decreeof the State Council [1993] No. 112
7	1993-07-07	NSC Document [1993] No.35
8	1993-09-02	Approval of the State Council [1993] No.122
9	1994-07-29	"Three major bailout policies"
10	1995-01-01	T+0 settlement is changed to T+1 settlement system
11	1995-05-17	Securities Regulatory Commission [1995] No. 62
12	1996-02-06	Securities Regulatory Commission [1996] No. 21
13	1996-10-31	Securities Regulatory Commission [1996] No. 7
14	1996-12-16	Highly alert to the current serious excessive speculation in the securities market and do 8 things
15	1997-03-03	Securities Regulatory Commission [1997] No. 7
16	1997-05-10	The State Council raised the stamp duty rate on securities transactions from 3‰ to 5‰
17	1997-05-21	NDRC Document [1997] No.16
18	1997-06-06	PBC Document [1997] No. 245
19	1998-04-22	Special treatment for listed companies with "abnormal financial status"
20	1998-06-21	The tax rate of stamp duty on securities transactions is reduced from 5‰ to 4‰
21	1998-12-29	OrderofthePresidentofthePeople’sRepublicofChina(No.12)
22	1999-07-29	Securities Regulatory Commission [1999] No. 94
23	2000-03-17	Securities Regulatory Commission [2000] No. 17
24	2000-04-03	The shares transferred are listed and circulated
25	2000-05-22	SFC Company [2000] No. 42
26	2000-06-17	SEC Market [2000] No. 10
27	2001-03-28	Order of the China Securities Regulatory Commission [No. 1]
28	2001-08-21	Securities Regulatory Commission [2001] No. 102
29	2001-08-31	SSE Law [2001] No. 8
30	2001-10-22	NDRC Document [2001] No.22
31	2002-05-21	Securities Regulatory Commission [2002] No. 54
32	2002-11-07	Order of the China Securities Regulatory Commission [No. 12]
33	2003-12-11	Order of the China Securities Regulatory Commission [No. 16]
34	2003-12-15	NDRC Document [2003] No.96
35	2004-02-01	Promulgation of A programmatic document to comprehensively deepen capital market reform
36	2004-05-17	The CSRC officially approved the establishment of the SME sector in Shenzhen Stock Exchange
37	2004-10-18	PBOC Announcement [2004] No. 12
38	2005-01-01	The regulation of "automatic queuing and limited number of reporters" is abolished
39	2005-05-08	Securities Regulatory Commission [2005] No. 66
40	2005-09-04	Securities Regulatory Commission [2005] No. 86
41	2006-05-15	SSEJ [2006] No.9、SZSC [2006] No. 54
42	2006-08-26	Order of the China Securities Regulatory Commission [No. 36]
43	2007-02-02	Order of the China Securities Regulatory Commission [No. 40]
44	2007-05-30	The stamp duty rate on securities transactions was raised from 1 ‰ to 3 ‰
45	2008-04-24	The tax rate of stamp duty on securities transactions was lowered from 3 ‰ to 1 ‰
46	2009-06-10	CSRCAnnouncement[2012]No.10
47	2012-06-08	The central bank cut interest rates for the first time in nearly three and a half years
48	2013-05-07	China Inter-bank Market Dealers Association Announcement [2014] No. 14
49	2013-12-13	Solicit public opinions on regulating the issuance and trading of preferred shares
50	2014-01-08	The IPO was closed for more than a year and then reopened
51	2014-11-17	The "Shanghai Hong Kong Stock Connect" opened
52	2015-01-08	SSE [2015] No. 5
53	2015-09-07	Finance and Taxation [2015] No. 101
54	2015-12-04	Shenzhen Stock Exchange and China Financial Exchange issued relevant regulations on index circuit breaker
55	2015-12-27	Authorizing a registration system for public offerings of shares to be listed on the SSE and SZSE
56	2016-01-01	New share issuance in the A-share market implemented under a new system
57	2016-06-27	"The official implementation of the "new three boards
58	2016-12-05	"Shenzhen-Hong Kong Stock Connect officially opened
59	2017-02-15	CSRCAnnouncement[2017]No.11
60	2017-06-21	Mingsheng announced that A-shares will be included in the MSCI emerging market index
61	2018-02-23	Adjust the term of the Securities Law of the People’s Republic of China
62	2018-05-31	After the closing, A-shares were formally included in the MSCI emerging market index
63	2018-09-27	FTSE Russell announced that A-shares will be included in its global stock index system
64	2018-11-16	SZSC [2018] No. 556
65	2019-01-30	CSRCAnnouncement[2019]No.2
66	2019-12-28	OrderofthePresidentofthePeople’sRepublicofChina(No.37)
67	2020-04-27	The GEM reform launched the pilot registration system.
68	2020-06-15	CSRC issued relevant rules and regulations on the GEM.
69	2021-01-04	The State Council executive meeting brought stamp duty on securities transactions into legal regulation.
70	2021-02-26	SFC Order [No.180]
71	2021-04-06	Main Board and the Small and Medium-sized Board were officially merged of Shenzhen Stock Exchange
72	2021-05-28	CSRC Announcement[2021]No.11
73	2021-07-15	SFC Order [No.186]
74	2022-05-20	SFC Order [No.197]
75	2022-06-24	SFC Order [No.200]

### Selection and description of stock market data

According to the need to test the weak efficiency of the stock market, we selected the stock price index as the research material. There are many stock price indexes in China’s stock market, among which the Shanghai Composite Index, the Shenzhen Composite Index and the Shanghai Shenzhen 300 Index are the most commonly used and most representative of the overall situation. The Shanghai Composite Index has been in existence since December 19, 1990, including all stocks listed on the Shanghai Stock Exchange, and based on the weighted acquisition of equity to share price of all stocks. According to the need to study the development of the entire stock market, we selected the Shanghai Composite Index and its yield as the research data. From December 20, 1990 to September 19, 2022, the data totaled 7758 trading days.

### Test method for weak efficiency of the stock market

In a weakly efficient stock market, stock price movements are independent of the relevant historical variables, so the time series data of stock price movements should conform to a random wandering pattern, and the price series should be not correlated with each other [36]. The run test is a statistical method used to test the randomness of a sample, and the one-sample run test can also be called the coherence test. Since we have adopted 75 major events, which are sufficient in number, so that other factors in different directions are offset, we can not consider other control variables. If the distribution of a feature of the sample is more disorderly and irregular, the more the randomness of the sample can be explained, and this feature is described by the number of trips. The number of trips is the total number of trips in a sequence, and the sum of the trips is the total number of trips in the sequence, denoted as *R*. For a fixed number of samples, the regularity of the sequence is too large or too small to satisfy the randomness. The critical value of *R* can be derived by querying the travel table to determine whether the sequence is random under small samples, and *R* approximately obeys normal distribution under large samples, so the statistic *Z* can be constructed to determine the randomness of the sequence:

$$Z=\frac{R-E(R)}{\sqrt{Var(R)}}$$ (1)

Where, *R* is the total number of runs; *E(R)* is the mean of the total number of runs; *Var(R)* is the variance of the total number of runs.


$$E(R)=\frac{2mn}{m+n}+1$$ (2)

$$Var(R)=\frac{2mn(2mn-m-n)}{{\left(m+n\right)}^{2}(m+n-1)}$$ (3)

In the above equation, *m* is the number of days the stock price has risen; *n* is the number of days the stock price has not risen; and *m+n* is the sample size (which, according to the description in the previous section, takes the value of 35 here). Run test shows that if the sequence is random and *R* follows the normal distribution, then the statistic *Z* follows the standard normal distribution. If the *Z* value falls within the interval corresponding to a certain significance of the standard normal distribution, the stock market is considered to be weakly efficient [37]. And another advantage of run test for explaining this problem is that it can realize random test of fixed interval data before and after any time point in Eviews programming. Thus, the change in the market’s weak effectiveness in the 35 trading days (fixed interval) before and after the introduction of a specific macro policy (a certain point in time) can be directly derived from the run test, so as to judge the effect of the implementation of a specific macro policy.

## Results and discussion

### Run test results general

**Fig 2:**
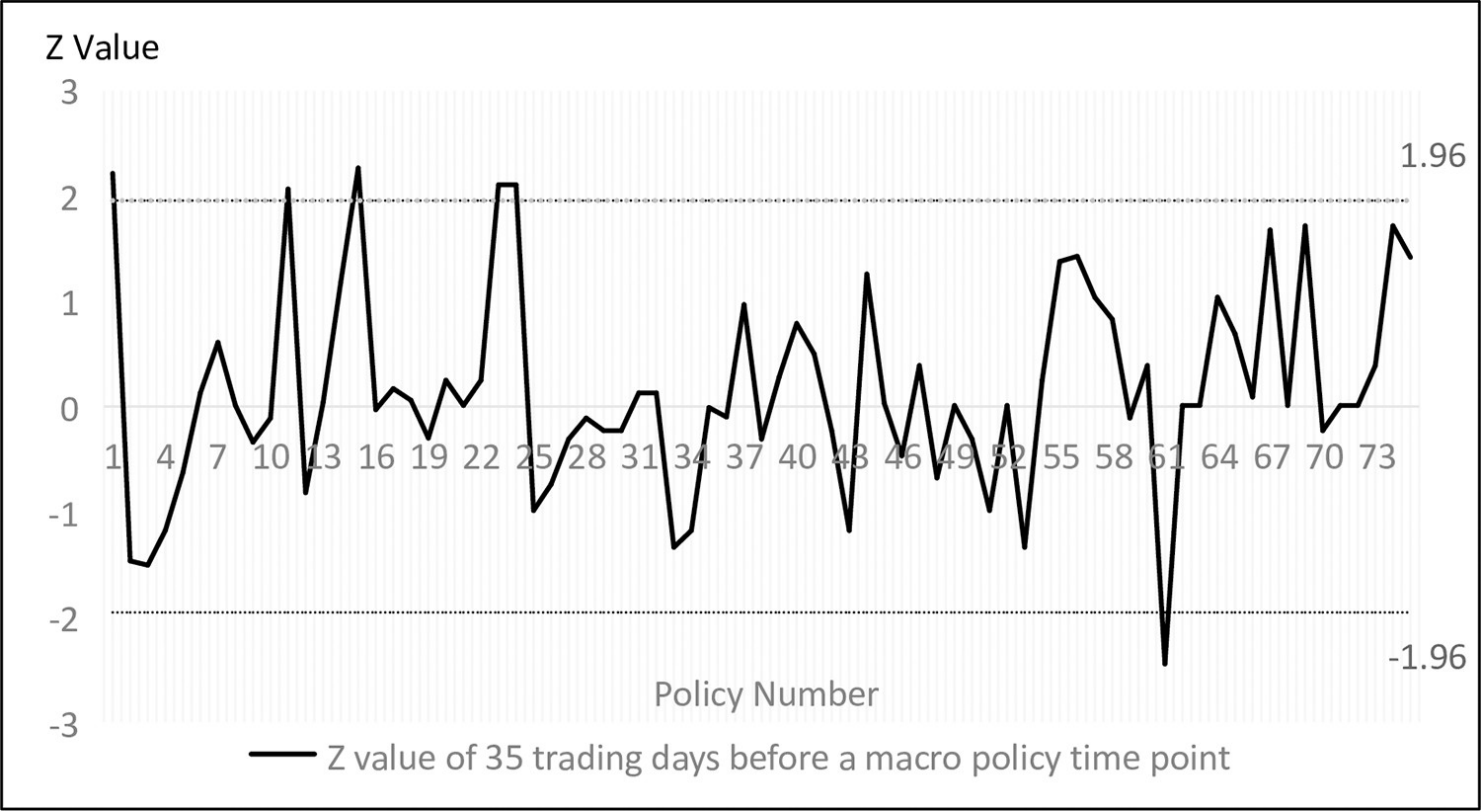
Run test results in 35 trading days before the policy event.

**Fig 3:**
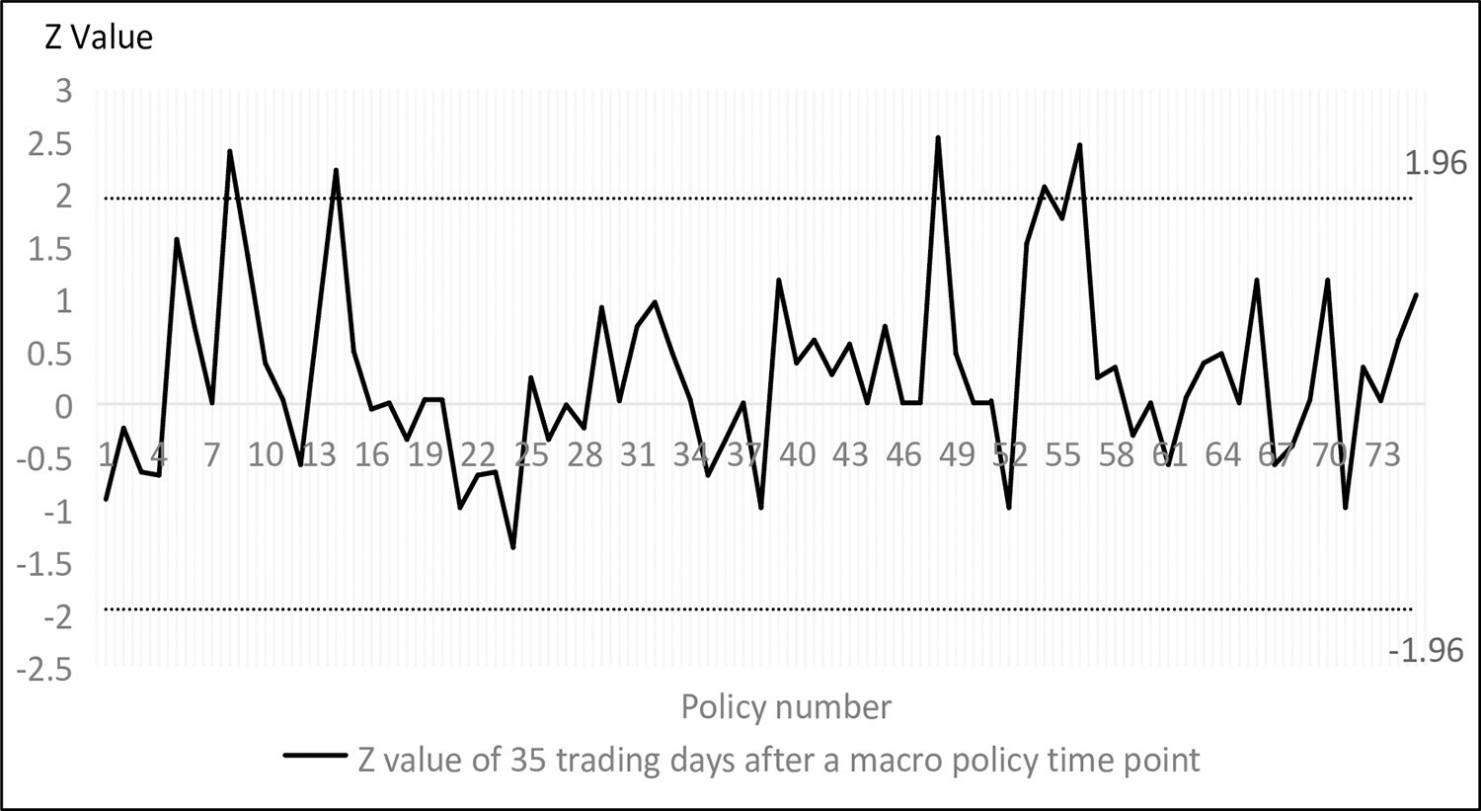
Run test results in 35 trading days after the policy event.

Figs 2 and 3 shows the results of the run test for all trading days, where the vertical coordinate refers to the *Z* value of the travel test for the 35 trading days before and after a certain point in time, through which the travel test values of market returns for the 35 trading days before and after the introduction of any macro policy can be easily selected to determine the changes in market effectiveness in the interval before and after. The *Z* values fall in (-1.96, 1.96) indicates that the stock market is weakly efficient in the 35 trading days before the time point, and the closer to zero, the greater the degree of weak efficiency. Conversely, a *Z* value outside (-1.96, 1.96) indicates that the stock market was non-weakly efficient in the 35 trading days prior to that point in time, and the farther away from the (-1.96, 1.96) interval, the greater the degree of non-weak efficiency.

As can be seen from Figs 2 and 3, the weakly effective degree of China’s stock market is in a relatively discrete state, but in general, with the continuous reform and development of China’s stock market, on the one hand, there is no longer the phenomenon of excessive non-weakly effective degree of the stock market in the initial years, and on the other hand, the number of trading days falling within the weakly effective range has also increased relatively gradually.

### Run test for specific policies

Whether macro policy affects the effectiveness of the securities market is mainly judged by the statistics *Z* of the return of 35 trading days before and after the introduction of a certain macro policy. According to the calculation results, the change of market effectiveness before and after the introduction of macro policy can be divided into 2 major categories and 4 subcategories, among which the 2 major categories are policies with positive linkage and policies with negative linkage. The positive linkage refers to the change of market effectiveness from non-weakly effective to weakly effective and the increase of weakly effective degree; the negative linkage refers to the change of market effectiveness from weakly effective to non-weakly effective and the decrease of weakly effective degree. Table 2 compares the changes in market effectiveness for the 35 trading days before and after the 75 macro policy events by serial number, and Table 3 provides descriptive statistics of the results.

**Table 2:** Macro-policy information and weak efficiency changes.

Policy serial number	Z value for the first 35 trading days	Z-value for the last 35 trading days	Weak effectiveness change situation	Policy serial number	Z value for the first 35 trading days	Z-value for the last 35 trading days	Weak effectiveness change situation
1	2.22	-0.91	Non-weak Effective to Weak effective	39	0.28	1.18	Weak effective weakening
2	-1.47	-0.23	Weak effective strengthening	40	0.79	0.39	Weak effective strengthening
3	-1.51	-0.65	Weak effective strengthening	41	0.50	0.61	Weak effective weakening
4	-1.18	-0.68	Weak effective strengthening	42	-0.23	0.28	Weak effective weakening
5	-0.63	1.57	Weak effective weakening	43	-1.18	0.57	Weak effective strengthening
6	0.13	0.74	Weak effective weakening	44	1.26	0.01	Weak effective strengthening
7	0.61	0.01	Weak effective strengthening	45	0.03	0.74	Weak effective weakening
8	0.01	2.41	Weak effective to non-weak effective	46	-0.47	0.01	Weak effective strengthening
9	-0.34	1.42	Weak effective weakening	47	0.39	0.03	Weak effective strengthening
10	-0.11	0.39	Weak effective weakening	48	-0.68	2.54	Weak effective to non-weak effective
11	2.07	0.04	Non-weak Effective to Weak effective	49	0.01	0.48	Weak effective weakening
12	-0.82	-0.58	Weak effective strengthening	50	-0.31	0.01	Weak effective strengthening
13	0.04	0.83	Weak effective weakening	51	-0.99	0.03	Weak effective strengthening
14	1.18	2.23	Weak effective to non-weak effective	52	0.01	-0.99	Weak effective weakening
15	2.27	0.50	Non-weak Effective to Weak effective	53	-1.34	1.53	Weak effective weakening
16	-0.03	-0.05	Weak effective weakening	54	0.25	2.07	Weak effective to non-weak effective
17	0.17	0.01	Weak effective strengthening	55	1.38	1.77	Weak effective weakening
18	0.06	-0.34	Weak effective weakening	56	1.43	2.47	Weak effective to non-weak effective
19	-0.30	0.04	Weak effective strengthening	57	1.04	0.25	Weak effective strengthening
20	0.25	0.03	Weak effective strengthening	58	0.83	0.35	Weak effective strengthening
21	0.01	-0.99	Weak effective weakening	59	-0.11	-0.30	Weak effective weakening
22	0.25	-0.68	Weak effective weakening	60	0.39	0.01	Weak effective strengthening
23	2.11	-0.65	Non-weak Effective to Weak effective	61	-2.45	-0.58	Non-weak Effective to Weak effective
24	2.11	-1.37	Non-weak Effective to Weak effective	62	0.01	0.06	Weak effective weakening
25	-0.99	0.25	Weak effective strengthening	63	0.03	0.39	Weak effective weakening
26	-0.74	-0.34	Weak effective strengthening	64	1.04	0.48	Weak effective strengthening
27	-0.31	-0.01	Weak effective strengthening	65	0.69	0.01	Weak effective strengthening
28	-0.11	-0.23	Weak effective weakening	66	0.09	1.18	Weak effective weakening
29	-0.23	0.92	Weak effective weakening	67	1.68	-0.58	Weak effective strengthening
30	-0.23	0.03	Weak effective strengthening	68	0.01	-0.40	Weak effective weakening
31	0.13	0.74	Weak effective weakening	69	1.72	0.04	Weak effective strengthening
32	0.13	0.97	Weak effective weakening	70	-0.23	1.18	Weak effective weakening
33	-1.34	0.48	Weak effective strengthening	71	0.01	-0.99	Weak effective weakening
34	-1.18	0.04	Weak effective strengthening	72	0.01	0.35	Weak effective weakening
35	-0.01	-0.68	Weak effective weakening	73	0.39	0.03	Weak effective strengthening
36	-0.10	-0.34	Weak effective weakening	74	1.72	0.61	Weak effective strengthening
37	0.97	0.01	Weak effective strengthening	75	1.42	1.04	Weak effective strengthening
38	-0.31	-0.99	Weak effective weakening				

**Table 3:** Statistics of macro-policy information and weak market efficiency changes.

Weak effective change situation	Number	Proportion
Non-weak effective to weak effective	6	8.00%
Weak effective strengthening	32	42.67%
Weak effective weakening	32	42.67%
Weak effective to non-weak effective	5	6.67%
Total number of samples	75	100%

### Discussion of results

Overall only about half of the stock market macro policy information has a positive linkage with stock market effectiveness, which include 6 times non-weak effective to weak effective and 32 times weak effective strengthening, and macro policies that have improved market effectiveness account for just over half of all policies. For example, the policy event 44 (on May 30, 2007, the stamp duty rate on securities transactions was raised from 1‰ to 3‰), which was introduced in the context of an obvious bubble in the stock market. Since then, the stock index has dropped from the highest point of 4335 on May 29 to the lowest point of 3858, and the market has returned to rationality.

Among these macro policies, 5 policies instead made the market ineffective from weak effective to non-weak effective, and 32 policies instead reduced the effectiveness of the market, which account for almost half of all policies. For example, the policy event 14 (the People’s Daily editorial pointed out that we should be highly vigilant against the serious excessive speculation and possible risks in the current securities market, and do eight jobs well). The introduction of this policy may have a certain degree of overcorrection and excessive intervention in the market, which reduces the effectiveness of the market.

**Fig 4:**
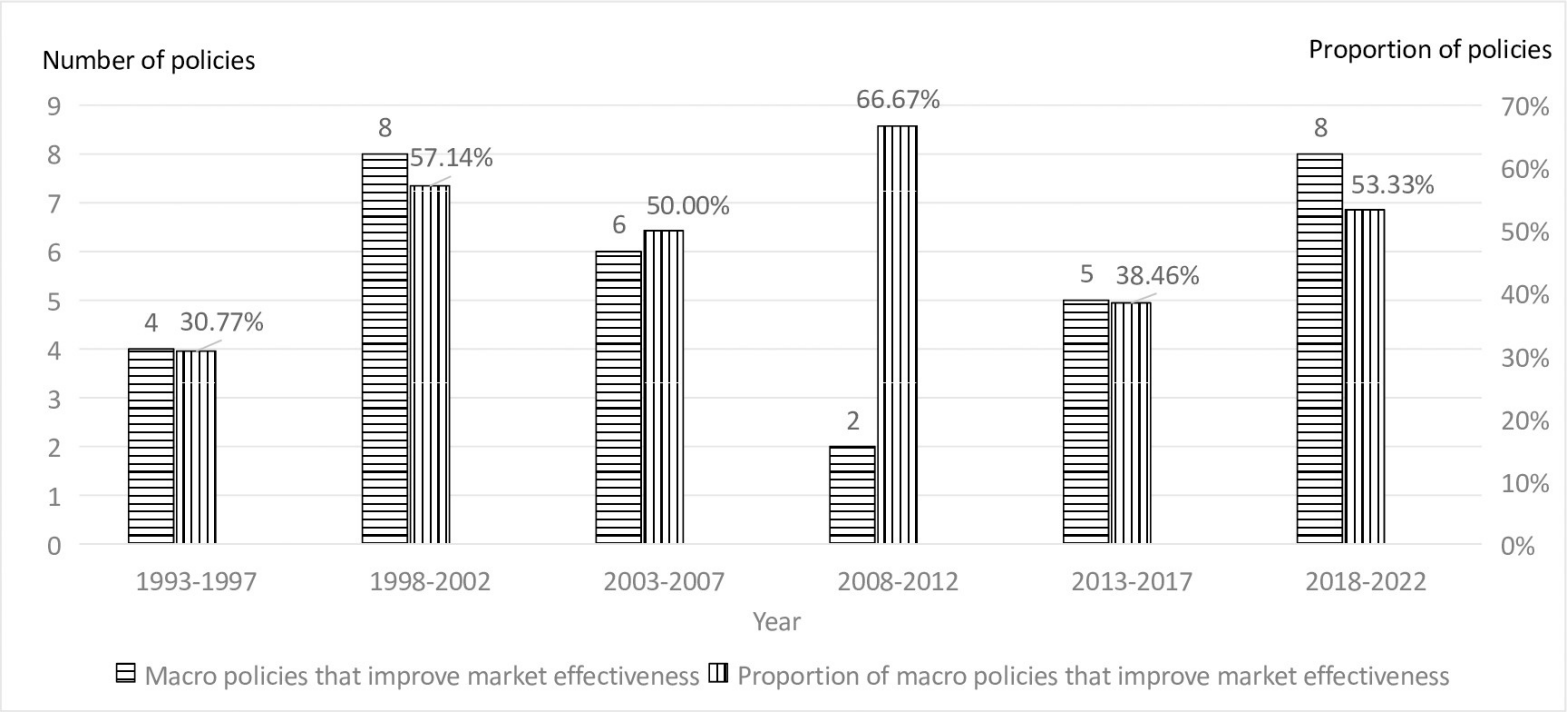
Time evolution chart of the macro policy effect in China’s stock market.

In general, the macro policies of China’s stock market do not always improve the effectiveness of the market, and almost half of the macro policies are ineffective. This phenomenon may be related to the growth of China’s stock market. In theory, with the passage of time and the improvement of policy formulation, the more recent macro policies should have more positive effects. Therefore, Fig 4 below takes five years as a group. Since 1993, the following 30 years have been divided into six groups to determine the effective proportion of macro policies within the six time periods (The data in 1992 were excluded because the newly established Chinese stock market was very low in marketization). It can be seen from Fig 3 that from the time scale of 30 years, the proportion of macro policies on China’s stock market that have improved the market effectiveness has not increased significantly, especially in the five years from 2013 to 2017. In those five years, the proportion of macro policies that have improved the market effectiveness is less than 40%. From the results of this analysis, the overall improvement of the macro policy of China’s stock market is not ideal.

From the above analysis, we regret to find that the effectiveness of China’s stock market macro policies has not improved significantly over time, and the prudence and effectiveness of China’s stock market macro policies are still worth improving. It can be seen that it is a long-term process to continue to improve various mechanisms of the securities market, improve the capital allocation ability of the securities market, reduce insider trading, and protect the interests of small and medium-sized investors [38].

## Conclusion

On the basis of sorting out the macro policy events in the past 31 years since the establishment of China’s stock market, based on the efficient market hypothesis and related assumptions, this paper studies the changes of weak market effectiveness 35 trading days before and after the introduction of 75 macro events through the method of run test, and concludes that the linkage between the macro policy information of China’s stock market and the effectiveness of the stock market is not always positive, and the nonlinear characteristics of the stock market are obvious, so the efficiency of the stock market still needs to be improved continuously and the stock market needs to be further improved. Therefore, suggestions are put forward for further standardizing and developing China’s stock market: First, the regulatory authorities should promote market data informatization, increase capital and technology investment, strengthen information disclosure mechanism, and improve market operation efficiency. In addition, strengthen the construction of big data platform, so that policy makers can timely understand the market operation, and provide a basis for the formulation of national macro policies. Second, adjust the role of the government and correctly grasp the appropriate policy intervention. The government should establish a dynamic market supervision mechanism, neither excessive intervention nor inaction, to realize the dynamic and institutionalized supervision of the stock market. Third,The degree of market effectiveness depends on whether the trading varieties are enough to deal with the stock market, so we should timely create stock market derivatives. Stock trading derivatives have the function of hedging, which can maintain the stability of stock prices to a certain extent, increase the return rate of assets, thus attracting more investment funds and increasing market activity.

At the same time, this study also explored a specific model of repeatable and improved information validity test of the stock market, which can continuously study the response of China’s stock market to relevant information and judge the degree of marketization of the development of the stock market. Although China’s stock market is mainly in the range of weak efficiency for a considerable period of time, it has not yet reached semi strong efficiency [39]. However, in the future research, we can continue to study the semi strong effectiveness of a certain interval before and after a macro policy event, so as to promote the existing research. In addition, we can also explore more methods to measure the effectiveness of the stock market in terms of measurement methods, measure the linkage between macro policy events and the effectiveness of the stock market from multiple dimensions, and avoid the possibility of using a single method to lead to low research reliability.

## Supporting information

S1 Data (CSV)
